# Pharmacokinetic-pharmacodynamic modeling of a highly potent and broadly neutralizing anti-CD4 trimeric nanobody to inhibit HIV-1 infection

**DOI:** 10.1128/spectrum.00805-25

**Published:** 2025-09-19

**Authors:** Xiaoqing Fan, Kangna Cao, Xilin Wu, Xiaoyu Yan

**Affiliations:** 1 Faculty of Medicine, School of Pharmacy, The Chinese University of Hong Kong, Hong Kong SAR, People's Republic of China; 2 Guangdong-Hong Kong-Macao Joint Laboratory for New Drug Screening, School of Pharmacy, The Chinese University of Hong Kong 26451, Hong Kong SAR, People's Republic of China; 3 Center for Public Health Research, Medical School, Nanjing University 117559, Nanjing, People's Republic of China; 4 Jiangsu Key Laboratory of Molecular Medicine, Medical School, Nanjing University 117559, Nanjing, People's Republic of China; 5 State Key Laboratory of Analytical Chemistry for Life Science, Nanjing University 644985 , Nanjing, People's Republic of China; University of Miami, Miami, Florida, USA

**Keywords:** target-mediated drug disposition, pharmacokinetic-pharmacodynamic modeling, anti-CD4 nanobody, Nb457-NbHSA-Nb457, HIV-1

## Abstract

Nb_457_ is a novel anti-CD4 nanobody derived from a CD4-immunized alpaca, exhibiting high potency and broad-spectrum activity against HIV-1. This study aims to use pharmacokinetic-pharmacodynamic (PK-PD) modeling that characterizes the time-course of Nb_457_ trimeric nanobody Nb_457_-Nb_HSA_-Nb_457_ and viral load in mice. The serum concentrations of Nb_457_-Nb_HSA_-Nb_457_ and HIV-1 load were modeled using a target-mediated drug disposition (TMDD) PK-PD model following intraperitoneal and subcutaneous administration of 400 µg in mice. All model parameters were estimated with high precision, with relative standard errors below 50%. The TMDD PK-PD model successfully captured the observed PK/PD profiles, demonstrating the strong therapeutic potential of Nb_457_-Nb_HSA_-Nb_457_ for HIV-1 treatment. Furthermore, the model was extrapolated to assess the feasibility of Nb_457_-Nb_HSA_-Nb_457_ for HIV-1 treatment in humans. The simulated viral growth trajectories at a dose of 20 mg/kg once every 2 days resulted in a downward trend in the slope of the viral trajectory, suggesting a failure to maintain replication and ultimately leading to viral suppression. Additionally, increasing the dosage or frequency of administration could further enhance the inhibition of viral replication. The simulated human PK-PD supports Nb_457_-Nb_HSA_-Nb_457_ as a promising anti-HIV-1 agent. This mechanistic TMDD PK-PD model provides a valuable tool to support the clinical development of Nb_457_-Nb_HSA_-Nb_457_ by enabling simulations of various dosing strategies to evaluate its efficacy and safety.

**IMPORTANCE:**

HIV-1 continues to pose a global health crisis, with millions of individuals depending on lifelong antiretroviral therapy, which faces significant challenges such as drug resistance and adherence issues. Nanobodies, which are small antibody fragments, present a promising alternative due to their high specificity, stability, and ease of production. Our study introduces Nb457-NbHSA-Nb457, a novel trimeric nanobody engineered to block HIV-1 entry by binding to CD4, the primary receptor for the virus. Using advanced pharmacokinetic-pharmacodynamic modeling, we predict the behavior of this therapy in humans, effectively bridging preclinical findings to clinical application. This research not only advances a new class of HIV therapeutics but also establishes a framework to expedite the development of nanobody-based drugs for infectious diseases, offering hope for simpler and more effective treatments to combat the pandemic.

## INTRODUCTION

Human Immunodeficiency Virus type 1 (HIV-1) infection continues to pose a significant global health challenge, with nearly 40 million people living with HIV-1 worldwide as of 2023 (1). Despite significant advances in antiretroviral therapy (ART), which has transformed HIV infection into a manageable chronic condition, current treatments are still hindered by several limitations, including virologic resistance, patient intolerance, adverse side effects, and high financial costs (2). Consequently, there is an urgent need for innovative therapeutic approaches to improve treatment outcomes and combat the ongoing HIV epidemic.

HIV fusion inhibitors, such as the membrane fusion inhibitor T20 (Fuzeon, enfuvirtide) and the C-C chemokine receptor type 5 (CCR5) blocker maraviroc (Selzentry), are currently utilized in combination therapy for HIV-1 infection. However, their clinical application has been limited due to low antiviral activity and the development of drug resistance (3). Recently, the primary host receptor CD4, which is essential for HIV-1 entry, has emerged as a promising target for HIV-1 treatment. In this context, potent broadly neutralizing antibodies (bNAbs) have gained attention as a promising alternative or complementary approach for HIV-1 immunotherapy (4). Notably, Ibalizumab, a humanized IgG4 antibody targeting CD4, became the first FDA-approved antibody drug for the treatment of multidrug-resistant HIV-1 in 2018, marking a significant milestone in HIV-1 therapy (5, 6). Despite its potential advantages for HIV-1 treatment, Ibalizumab is constrained by its relatively narrow breadth and potency, high production costs, and significant immunogenicity associated with long-term administration.

Nanobodies, a unique class of antibody fragments derived from camelid heavy-chain-only antibodies and consisting of a single monomeric variable antibody domain have emerged as promising therapeutic agents due to their small size, high specificity, stability, and ease of production. Among their various potential applications, targeting CD4 with nanobodies represents a novel and innovative approach for HIV treatment. By blocking the interaction between HIV-1 and CD4, nanobodies can effectively neutralize the virus and prevent the infection of host immune cells. We have successfully developed a novel anti-CD4 nanobody, Nb_457_, and demonstrated its potent activity in neutralizing HIV-1 (7). To further optimize its therapeutic potential, a trimeric nanobody, Nb_457_-Nb_HSA_-Nb_457_ (~40 kDa) was developed to address potential side-effects associated with the Fc fusion tag and to extend the *in vivo* half-life of Nb_457_ (7). The binding of Nb_457_-Nb_HSA_-Nb_457_ induces a conformational alteration of CD4, impairing the ability of HIV-1 gp120 to bind to CD4, thereby preventing viral entry into host cells. These preclinical findings underscore the potential of Nb_457_-Nb_HSA_-Nb_457_ as a promising therapeutic candidate for HIV-1 treatment.

To advance Nb_457_-Nb_HSA_-Nb_457_ toward clinical application, mathematical modeling is required to quantify its pharmacokinetics (PK) and the pharmacodynamics (PD) of HIV-1 dynamics *in vivo*. Such modeling approaches have been widely utilized and have significantly advanced our understanding of HIV-1 dynamics and the interactions between the virus and CD4+ cells in infected patients (8–10). These approaches can provide valuable insights to guide the clinical development of Nb_457_ for HIV-1 treatment and/or prevention.

In this study, we aim to develop a target-mediated drug disposition (TMDD) PK-PD model to characterize the PK/PD profiles of the anti-CD4 trimeric nanobody Nb_457_-Nb_HSA_-Nb_457_ and assess its feasibility for HIV-1 treatment in humans. This model will provide critical insights into the nanobody’s efficacy, optimal dosing strategies, and potential for clinical translation, paving the way for the development of a novel therapeutic option for HIV-1 infection.

## MATERIALS AND METHODS

### Data

The data used in this study were derived from our previous study (Table S1) (7). The anti-HIV activity of Nb_457_-Nb_HSA_-Nb_457_ was evaluated in a humanized mouse model (NDG-HuPBL) infected with 10 ng of P24 HIV-1_CH058_ (7). Due to the limited blood volume available in mice, PK and PD studies were conducted separately. For the PK study, the infected NDG-HuPBL mice (*n* = 4, female) were treated with 400 µg of Nb_457_-Nb_HSA_-Nb_457_ per mouse via intraperitoneal (IP) or subcutaneous (SC) on day 1 post-infection. Blood samples were drawn at 0 min, and 1, 4, 8, 12, 24, 72, and 120 h after the dose. The concentration of Nb_457_-Nb_HSA_-Nb_457_ in serum was measured using an in-house ELISA as described in our previous study (7). Briefly, high protein-binding ELISA plates (Cat#: 9018, Corning) were coated with protein at 0.5 µg/mL (100 µL per well) and incubated either overnight at 4°C or for 2 h at 37°C. After washing, plates were blocked with 5% non-fat milk in PBS at 37°C for 1 h. Serially diluted anti-sera or purified antibody (100 µL) was added to each well and incubated at 37°C for 90 min. Following additional washes, plates were treated with HRP-conjugated secondary antibodies: goat anti-human IgG (1:10,000 dilution, Cat#: 109-035-088, Jackson ImmunoResearch) or goat anti-llama IgG (H + L) (1:10,000 dilution, Cat#: NB7242, Novus) for 1 h at 37°C. Subsequently, the TMB substrate (Sigma) was added and allowed to react at 37°C for 10 min. The reaction was stopped by adding 10 µL of 0.2 M H₂SO₄, and optical densities were measured at 450 nm using the Infinite 200 instrument (Tecan, Ramsey, MN, USA). For the PD study, the infected NDG-HuPBL mice (*n* = 4, female) were treated with 400 µg of Nb_457_-Nb_HSA_-Nb_457_ per mouse via intraperitoneal (IP) on day 1 post-infection, followed by subcutaneous (SC) injection on days 3, 5, and 7 after HIV-1_CH058_ challenge. Blood samples for PD analysis were drawn on weeks 1, 2, 3, and 4 post-infection. The viral load was monitored by quantitative polymerase chain reaction (qPCR). Meanwhile, Ibalizumab (~150 kDa) was used as a positive control and administered using the same dosing regimen. The experimental procedures for this research received approval from the Center for Public Health Research at the Medical School of Nanjing University. All mice were treated in accordance with the China Ethical Guidelines for the Welfare of Laboratory Animals (GB 14925-2010). Additionally, protocols involving animals infected with HIV adhered to the Guidelines set forth by the Hubei Laboratory Animal Science Association (Approval number: WIVA11202202).

### Development of mechanism-based PK-PD modeling

**Fig 1:**
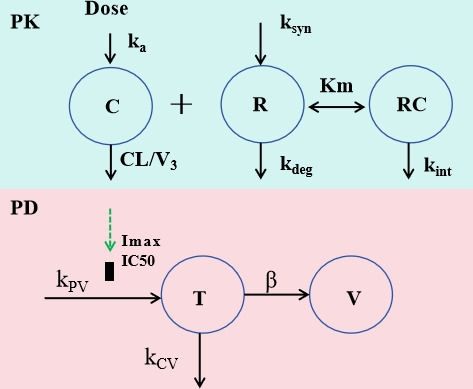
Schematic diagram of the TMDD PK-PD model for anti-CD4 nanobody on viral dynamic. Symbols are defined in method section.

To quantify the effects of Nb_457_-Nb_HSA_-Nb_457_ and Ibalizumab in mice with HIV infection, a mechanism-based target-mediated drug disposition (TMDD) PK-PD model was developed. The model structure is shown in Fig. 1.

### PK model

The Nb_457_-Nb_HSA_-Nb_457_ and Ibalizumab PK data in mice following IP and SC doses (400 µg) were fitted to a TMDD PK model assuming quasi-equilibrium (QE) (8, 11). The differential equations are as follows (eqs 1 to 5-5):


(1) $$\frac{dA_{1}\left(t\right)}{dt}=-K_{a\left(IP\right)}\cdot A_{1}\left(t\right)\;$$

(2) $$\frac{dA_{2}\left(t\right)}{dt}=-K_{a\left(SC\right)}\cdot A_{2}\left(t\right)$$

(3) $$\frac{dA_{3}\left(t\right)}{dt}=k_{a\left(IP\right)}\cdot A_{1}\left(t\right)+k_{a\left(SC\right)}\cdot A_{2}\left(t\right)-CL\cdot C_{3}-\left(A_{3}\left(t\right)-C_{3}\cdot V_{3}\right)\cdot k_{int}\;$$

(4) $$\frac{dA_{4}\left(t\right)}{dt}=k_{syn}-\left(k_{int}-k_{deg}\right)\cdot \left(\frac{A_{3}\left(t\right)}{V_{3}}-C_{3}\right)-K_{deg}\cdot A_{4}\left(t\right)\;where\;A_{4}\left(0\right)=R_{TOT0}$$

(5) $$C_{3}=\frac{1}{2}\times (\frac{A_{3}(t)}{V_{3}}-A_{4}(t)-k_{M}+\sqrt{{\left(\frac{A_{3}(t)}{V_{3}}-A_{4}(t)-K_{M}\right)}^{2}+4\cdot K_{M}\cdot \frac{A_{3}(t)}{V_{3}}}$$

where *A*_1_(*t*), *A*_2_(*t*), and *A*_3_(*t*) are the amounts of Nb_457_-Nb_HSA_-Nb_457_ or Ibalizumab in the IP, SC, and central compartments, respectively. Initial conditions for *A*_1_, *A*_2_, and *A*_3_ were zero. *k*_a(IP)_ and *k*_a(SC)_ are the absorption rates of Nb_457_-Nb_HSA_-Nb_457_ or Ibalizumab at IP and SC, respectively; *V*_3_ is the volumes of the central compartment; *CL* is the linear clearance from the central compartment; *k*_int_ denotes the rate constant of drug-receptor complex internalization, while *k*_deg_ represents the degradation rate of free receptor. To simplify the model and reduce the risk of overparameterization, *k*_int_ was assumed to be equal to *k*_deg_. *k*_syn_ is the endogenous synthesis rate of target; *K*_M_ is the drug-receptor complex equilibrium dissociation constant; *R*_TOT0_ is the estimated amount of free receptor at time 0.

### PD model

The PD model was described by using a viral dynamic model, which was modified from previous publications (12, 13). The model included a pool of uninfected target cells that are available for the HIV-1 to infect, subsequently replicate, and be released to infect more cells. The differential equations are as follows (eqs 6 and 7):


(6) $$\frac{dA_{5}\left(t\right)}{dt}=k_{PV}\cdot A_{5}\left(t\right)\cdot \left(1-\frac{I_{max}\cdot C_{3}}{SC_{50}+C_{3}}\right)-k_{CV}\cdot A_{5}\left(t\right)\;$$

(7) $$\frac{dA_{6}\left(t\right)}{dt}=\beta \cdot \left(\lambda \cdot A_{5}\left(t\right)-A_{6}\left(t\right)\right)$$

where *A*_5_(*t*) and *A*_6_(*t*) are the amounts of T cells and HIV-1 compartments, respectively. Here, the T cells compartment is a transit compartment for model fitting, only viral load data were fitted in the model. *k*_PV_ is the viral infection rate, *k*_CV_ is the death rate of infected cells, *β* is the rate constant for HIV release, while *λ* is the ratio of infected T cells to HIV viral load. Initial conditions were estimated for T cells and zero for HIV-1. *I*_max_ is the maximum inhibitory effect of the drug on blocking HIV-1 infection, and SC_50_ is the concentration of the drug that induces a half-maximum effect.

Given the limited sample size (*n* = 4) available for the sparse-sample study, a naïve pooled data approach was employed. In this method, data from all individuals were treated as if they originated from a single unique individual. Consequently, the interindividual variabilities (IIVs) for the fixed-effect parameters were not preserved.

The residual variability was characterized using a proportional error model:


(8) $$Y_{ij}={\hat{Y}}_{ij}\cdot \left(1+\varepsilon_{1}\right)$$

where *Y*_*ij*_ represents the observation of individual *i* at time *t*_*j*_, *Ŷ*_*ij*_ is the corresponding model prediction, and *ε*_1_ is assumed to be an independent and normally distributed random variable with a mean of zero and standard deviation (*σ*).

The ordinary differential equations were solved using the ADVAN13 subroutine, and parameter estimation was performed using the first-order conditional estimation method with interaction (FOCEI) algorithm.

### Model evaluation and model-based simulations

Model selection and evaluation were conducted based on the objective function value, parameter precision, and visual assessment of graphical diagnostics. Diagnostic plots were generated using R for initial visual inspections, including observed values vs population predicted values (PRED), individual predicted values (IPRED), conditional weighted residuals (CWRES) vs PRED, and CWRES vs time. The final model underwent additional evaluation through simulations of the PD time course using the estimated parameters. The observed data were then overlaid with the simulated data.

### Extrapolation of the mouse model to humans

To verify the accuracy and reliability of the extrapolation, we first applied the Ibalizumab model to humans, as published PK data were available for validation (14). Relevant information (including dosing regimen and measurements) obtained from the literature for validation is summarized in Table S2. However, due to the limited availability of PD viral dynamic data the different clinical scenarios, the PD extrapolation could not be validated. Allometric scaling was applied based on parameters derived from the TMDD model. The allometric exponent for the volume of distribution (*V*3) was set to 1, while the exponent for linear clearance (CL) was set to 0.85. It has been suggested that the exponent 0.85 provided the best prediction of human *CL* from the mouse. The *CL* and first-in-human dose of monoclonal antibodies (mAbs) were predicted reasonably well by a single-species mouse (15–17). In the TMDD model, all other human CD4-targeted parameters were assumed to be equivalent to those observed in mice. For the PD model, all the human viral dynamic-related parameters were assumed same as the mouse parameters because the experiments were performed in humanized mouse models. Then, to evaluate the feasibility of Nb_457_-Nb_HSA_-Nb_457_ for HIV-1 treatment in humans, the extrapolated model was utilized to simulate human PK-PD following SC administration.

### Software

PK/PD model analysis was conducted using NONMEM 7.5 (Icon Development Solutions, Ellicott City, MD, USA), with support from Perl-speaks-NONMEM (version 4.9.6, https://uupharmacometrics.github.io/PsN/). The ordinary differential equations were solved using the ADVAN14 subroutine, and parameter estimation was performed using the first-order conditional estimation method with interaction (FOCEI) algorithm. Data comparisons and visualizations were carried out using R (version 4.3.3, www.r-project.org).

## RESULTS

### Data collection of the PKPD model

**Fig 2:**
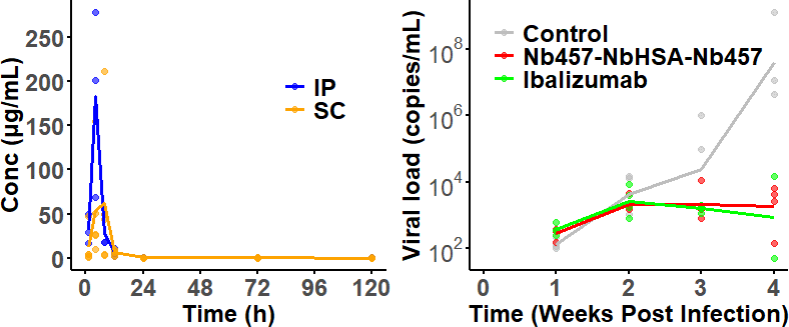
Time course profiles of Nb_457_-Nb_HSA_-Nb_457_ serum concentrations (Left) after IP (blue line) and SC (orange line) injections of 400 µg, and the plasma viral load dynamic profiles (Right) in three groups of NDG-HuPBL mice assessed through qRT-PCR. Negative control group (gray line), (green line), Nb457-NbHSA-Nb457 (red line), and Ibalizumab (green line). Each line represents average data from one group of mice (*n* = 4 in each group). Data from an individual mouse is represented by a dot. The data were derived from our previous study (7).

The observed data for Nb_457_-Nb_HSA_-Nb_457_ and Ibalizumab across different administration routes are shown in Fig. 2 and Fig. S1. Further details about the study are available in our previous study (7). Intraperitoneal (IP) administration of Nb_457_-Nb_HSA_-Nb_457_ led to a rapid peak serum concentration (*C*_max_) of 175.5 µg/mL, accompanied by a short half-life (*t*_1/2_) 7.47 h. In contrast, subcutaneous (SC) administration resulted in a longer time to reach *C*_max_ (67.04 µg/mL) but yielded a more extended half-life of 27.71 h. For Ibalizumab, the PK trends mirrored those of Nb_457_-Nb_HSA_-Nb_457_ for both administration routes, with *C*_max_ values of 315.3 µg/mL for IP and 251.5 µg/mL for SC, and half-lives of 24.75 h and 34.31 h, respectively (Fig. S1). The viral load in the control group exhibited a progressive increase, while both the Nb_457_-Nb_HSA_-Nb_457_ and Ibalizumab treatment groups demonstrated substantial reductions in viral loads at 3 and 4 weeks post-infection, indicating robust inhibitory effects of both treatments on HIV-1 replication *in vivo*.

### The model fits the literature data well

The mechanism-based TMDD PK-PD model is depicted in Fig. 1. TMDD models are commonly employed to describe the PK of drugs whose distribution and/or clearance are affected by target interactions, particularly in cases of high binding affinity and limited target capacity. These models account for the turnover of the capacity term and provide a mechanistic representation of drug-target kinetics (18). In this model, the quasi-equilibrium (QE) assumption was applied to simplify the model and reduce the number of parameters. A sequential modeling approach was implemented, where typical PK parameters derived from the PK model were subsequently used to drive the PD effects. The PK parameters estimated through TMDD modeling are presented in Table 1, while the PD parameters obtained via sequential fitting are shown in Table 2. The IC_50_ values for each drug were fixed based on experimentally observed data because it would be inaccurate to estimate IC_50_ with only one dose level since PD is nonlinear and its thorough evaluation requires a minimum of two to three doses (7, 19). Additionally, Nb_457_-Nb_HSA_-Nb_457_ is a highly potent and broadly neutralizing anti-CD4 trimeric nanobody, the IC_50_ values are quite similar among different HIV-1 strains. The IC_50_ values against five distinct live HIV-1 strains: HIV-1_BAL_, HIV-1_CH058_, HIV-1_THRO_, HIV-1_CH042_, and HIV-1_CH198_, were 0.3585, 0.0341, 0.0277, 0.0340, and 0.0195 µg/mL, respectively (7). Therefore, the results should be applied to other HIV-1 strains. All PK and PD parameters were estimated with high precision, with relative standard errors (RSE) below 50% (Tables 1 and 2).

**TABLE 1:** Model estimates of the PK parameters of Nb_457_-Nb_HSA_-Nb_457_ and Ibalizumab together with their relative standard errors (RSE)

Drug	Parameters (Units)	Description	Estimate	%RSE^*a*^
Nb_457_-Nb_HSA_-Nb_457_	*CL* (mL/h)	Clearance	0.69	33.21
	*V*_3_ (mL)	Volume of distribution of the central compartment	0.73	12.49
	*k_a_*_(IP)_ (1/h)	Absorption rate after intraperitoneal injection	0.46	2.02
	*k_a_*_(SC)_ (1/h)	Absorption rate after subcutaneous injection	0.37	7.03
	RTOT_0_ (μg/mL)	Baseline total receptor	174.7	12.09
	*K*_M_ (μg/mL)	Michaelis constant	0.14	19.25
	*k*_int_ (1/h)	Internalization rate constant	0.0027	42.4
	*ω*_CL_	Inter-individual variability of *CL*	0.87	27.73
	*ω*_V3_	Inter-individual variability of *V*_3_	0.082	42.75
	*σ*_1_	Proportional error of Nb_457_-Nb_HSA_-Nb_457_	0.82	11.75
	*σ*_2_	Additive error of Nb_457_-Nb_HSA_-Nb_457_	0.04	10.2
Ibalizumab	*CL* (mL/h)	Clearance	0.024	20.29
	*V*_3_ (mL)	Volume of distribution of the central compartment	0.54	35.27
	*Q* (mL/h)	Tissue distribution clearance	1.86	35.27
	*V*_4_ (mL)	Volume of distribution of the peripheral compartment	0.12	14.25
	*k_a_*_(IP)_ (1/h)	Absorption rate after intraperitoneal injection	0.26	46.85
	*k_a_*_(SC)_ (1/h)	Absorption rate after subcutaneous injection	0.22	58.57
	RTOT_0_ (μg/mL)	Baseline total receptor	11.73	249.6
	*K*_M_ (μg/mL)	Michaelis constant	12.73	308.5
	*k*_INT_ (1/h)	Internalization rate constant	0.0036	1121
	*ω*_CL_	Inter-individual variability of *CL*	0.29	22.95
	*ω*_V3_	Inter-individual variability of *V*_3_	0.68	40.53
	*σ*_1_	Proportional error of Nb_457_-Nb_HSA_-Nb_457_	0.053	8.69

^*a*^ RSE for *ω* and *σ* are reported on the approximate S.D. scale (standard error/variance estimate)/2. The PK data of Ibalizumab were fitted to a two-compartment disposition model. The other model structure and parameters are the same as Nb_457_-Nb_HSA_-Nb_457_.

**TABLE 2:** Model estimates of the PD parameters of Nb_457_-Nb_HSA_-Nb_457_ and Ibalizumab together with their relative standard errors (RSE)

Drug	Parameters (Units)^*a*^	Description	Estimate	%RSE^*b*^
Nb_457_-Nb_HSA_-Nb_457_	*k*_PV_ (1/h)	Viral infection rate	0.041	14.1
	*k*_CV_ (1/h)	Death rate of infected cells	0.025	22.72
	*β* (1/h)	Rate constant for HIV release	8.12 × 10^−7^	21.1
	*λ*	Ratio of infected T cells to HIV viral load	0.81	36.1
	BASE_T_ (cells/mL)	Baseline CD4^+^ T cells	814.2	44.2
	*I*_max_	Maximum inhibitory effect of Nb_457_-Nb_HSA_-Nb_457_ on blocking HIV-1 infection	1.963	16.24
	IC_50_ (μg/mL)	The concentrations that induce a half-maximum effect	0.0341	–^*c*^
	*σ*_3_	Proportional error of viral load	0.21	14.5
Ibalizumab	*k*_PV_ (1/h)	Viral infection rate	0.027	12.68
	*k*_CV_ (1/h)	Death rate of infected cells	0.0072	38.42
	*β* (1/h)	Rate constant for HIV release	2.09 × 10^−7^	58
	*λ*	Ratio of infected T cells to HIV viral load	2.001	38.67
	BASE_T_ (cells/mL)	Baseline CD4^+^ T cells	1,225	36.38
	*I*_max_	Maximum inhibitory effect of Ibalizumab on blocking HIV-1 infection	0.42	51.79
	SC_50_ (μg/mL)	The concentrations that induce a half-maximum effect	0.1449	–^*c*^
	*σ*_3_	Proportional error of viral load	0.94	23.09

^*a*^ The PK parameters were fixed at their estimated values.

^*b*^ RSE for *ω* and *σ* are reported on the approximate S.D. scale (standard error/variance estimate)/2.

^*c*^ The IC_50_ was fixed to the observed value.

**Fig 3:**
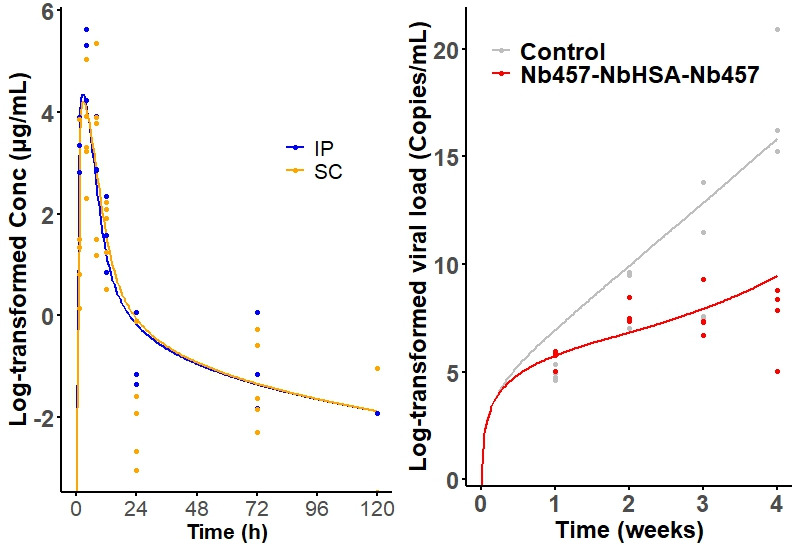
Visual predictive checks (VPC) for the Nb_457_-Nb_HSA_-Nb_457_ PK (left) and PD model (right). The solid lines represent the median of the model predictions, and the dots represent the observed data.

Goodness-of-fit (GOF) diagnostic plots for the final PK and PD models revealed a random scatter around the identity line, indicating an absence of systematic bias (Fig. S2 to S5). The model successfully captured both the overall trends and observed variability, confirming its suitability for describing the data. Visual predictive checks further demonstrated that the model effectively described the PK profiles and PD responses of Nb_457_-Nb_HSA_-Nb_457_ (Fig. 3) and Ibalizumab in HIV-1-infected mice (Fig. S6).

### Extrapolation of the PK/PD Model to Humans

To evaluate the feasibility of developing Nb_457_-Nb_HSA_-Nb_457_ as a therapeutic agent for the treatment of HIV-1, the established TMDD PK-PD model was scaled from mice to humans. To ensure the accuracy and validity of the extrapolation, we first applied the Ibalizumab model to humans, leveraging the availability of published PK data for validation. The allometry exponent for the volume of distribution was set to 1, predicting a human central compartment volume of distribution of 26.9 mL/kg. For linear clearance, the exponent was established at 0.85, resulting in a projected human linear clearance of 0.35 mL/h/kg. As illustrated in Fig. S7 and S8, the model successfully predicted the PK profiles of Ibalizumab following multiple-dose regimens (Fig. S7). Moreover, nearly all model-estimated concentrations varied within a twofold error margin compared to the observed data (Fig. S8), demonstrating acceptable predictive accuracy.

**Fig 4:**
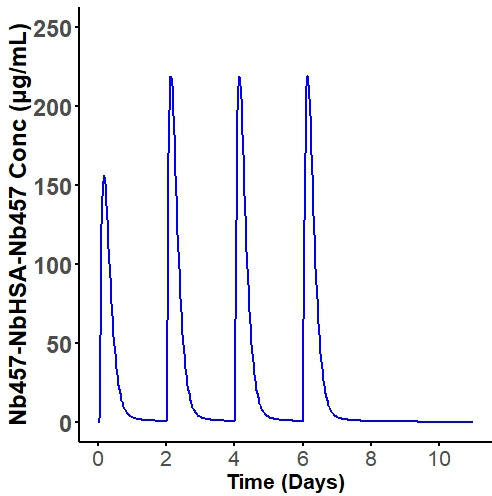
Simulated human serum concentration-time profiles of Nb457-NbHSA-Nb457 following 20 mg/kg once every 2 days for four doses.

For Nb_457_-Nb_HSA_-Nb_457_, the scaled human central compartment volume of distribution was 36.26 mL/kg and human linear clearance of 10.21 mL/h/kg. Considering that the SC route is the most likely administration method for protein drugs in clinical settings (8), only the PK profile following SC administration was simulated. As illustrated in Fig. 4, the human PK of Nb_457_-Nb_HSA_-Nb_457_ after SC administration was simulated based on model parameters derived from the one-compartment TMDD model. A dosing regimen of 20 mg/kg once every 2 days was used to simulate the PK profile, revealing slight accumulation (Fig. 4), which aligns with previous findings indicating that continuous protein administration results in modest accumulation (14, 20).

**Fig 5:**
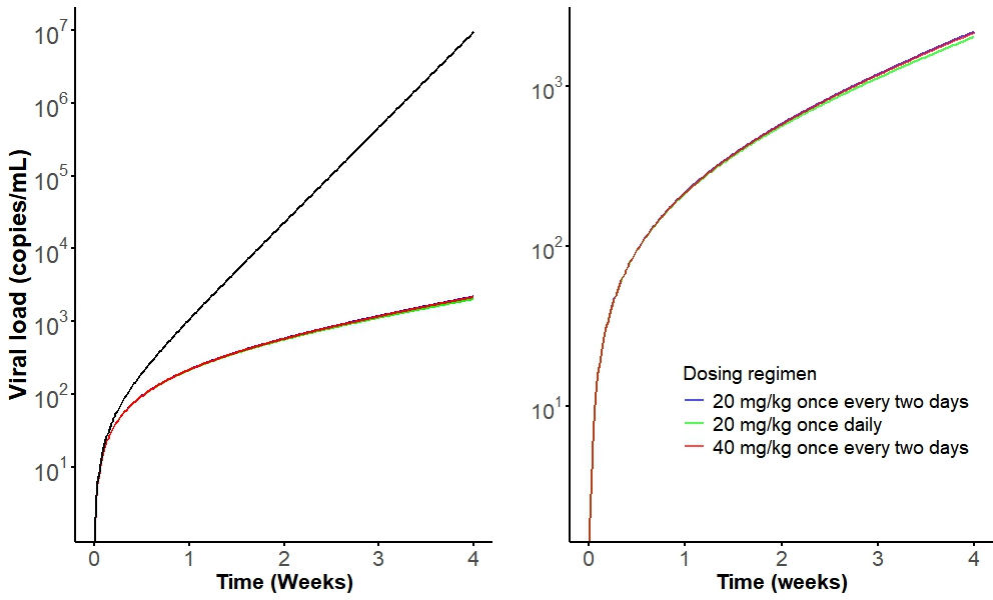
Simulated human viral load profiles under different dosing regimens. The left figure shows log-transformed viral load profiles with control group (black) and treatment groups (blue, green, and red). The right figure shows the viral load profiles in the three treatment groups. The drug administration lasted for 1 month (14 doses for once every 2 days and 28 doses for once daily).

The simulated viral growth trajectories at a dose of 20 mg/kg once every 2 days mimic the HIV-1 replication profile observed in raw data (Fig. 5). Treatment with Nb_457_-Nb_HSA_-Nb_457_ resulted in a downward trend in the slope of the viral trajectory, suggesting a failure to maintain replication and ultimately leading to viral suppression. Additionally, increasing the dosage or frequency of administration could further enhance the inhibition of viral replication (Fig. 5). Collectively, the simulated human PK-PD data support the potential of Nb_457_-Nb_HSA_-Nb_457_ as a promising anti-HIV-1 agent.

## DISCUSSION

The development of effective therapies for HIV-1 infection remains a significant challenge. Despite significant advancements in ART, which has effectively transformed HIV into a manageable chronic condition, the ongoing pandemic, and the limitations of current treatments such as drug resistance, side effects, and the economic burden of lifelong therapy necessitate the development of novel therapeutic strategies (1, 21, 22). Nanobodies have emerged as a promising alternative to traditional antibodies in drug development due to their unique properties, including small size, high thermal stability, and exceptional specificity. A previous study developed an anti-CD4 trimeric nanobody, Nb_457_-Nb_HSA_-Nb_457_, which demonstrated a favorable safety profile and achieved complete neutralization of various HIV-1 strains, with a maximum inhibition rate of 100% (7). These findings highlight the potential of Nb_457_-Nb_HSA_-Nb_457_ as a promising therapeutic candidate for HIV-1. However, further studies are required to quantify the exposure-response relationship to guide the dosing regimen in humans.

PK/PD modeling based on preclinical data offers a valuable approach to guiding drug development for clinical applications (10). Using data from *in vitro* studies and mouse models, we developed a mechanistic TMDD PK-PD model to predict the dosing regimen of Nb_457_-Nb_HSA_-Nb_457_ in humans. The TMDD PK model characterizes the plasma concentration of Nb_457_-Nb_HSA_-Nb_457_, as well as its interaction with the target receptor. In the TMDD model of both Nb_457_-Nb_HSA_-Nb_457_ and Ibalizumab, *k*_int_ was assumed to be equal to *k*_deg_. This assumption not only reduced the number of model parameters, mitigating the risk of overparameterization, but also aligned with the mechanism of action of the two drugs, which induces CD4 conformational changes rather than stimulating CD4 internalization. Consequently, Nb_457_-Nb_HSA_-Nb_457_ and Ibalizumab followed the normal turnover rate of the CD4 receptor. The proposed TMDD PK model successfully captured the PK of Nb_457_-Nb_HSA_-Nb_457_ and Ibalizumab, providing a robust framework for predicting drug concentrations under various assumptions.

To develop the PK/PD model, several assumptions were used due to limited data availability. For simplicity, we assumed negligible effects of immune cells (e.g., ingress/egress of blood or immune cells) on viral clearance and a constant rate of viral production and growth. These assumptions represent a worst-case scenario in which reductions in viral growth are attributed solely to the drug’s effects, excluding any contribution from immune responses. Compared to Ibalizumab, Nb_457_-Nb_HSA_-Nb_457_ demonstrates a greater absorption rate (Table 1) and superior efficacy in neutralizing HIV-1, the IC_50_ value of is Nb_457_-Nb_HSA_-Nb_457_ 10-folder greater than Ibalizumab (Table 2) (7). However, its shorter half-life necessitates higher dosing intensity to maintain therapeutic concentrations. Notably, SC administration resulted in a longer half-life compared to IP administration despite achieving lower peak concentrations. Given Nb_457_-Nb_HSA_-Nb_457_’s high potency, with an IC_50_ value of 0.0341 µg/mL, SC administration is predicted to sustain drug concentrations at target sites for an extended duration, effectively blocking HIV-1 infection. Based on these findings, SC administration was used to simulate the human PK-PD profile to ensure sufficient active drug concentrations over time within target cells.

To evaluate the feasibility of developing Nb_457_-Nb_HSA_-Nb_457_ as a therapeutic agent for the treatment of HIV-1 in humans, we simulated its human PK-PD profiles following SC administration using parameters derived from the TMDD PK-PD model and allometric scaling. We used an allometric scaling approach based on mouse PK data with an exponent of 0.85 for clearance and an exponent of 1 for volume of distribution. The allometric scaling approach was based on mouse PK data, applying an exponent of 0.85 for clearance and 1 for the volume of distribution. This method is supported by multiple studies demonstrating that an allometric scaling exponent of 0.85 for clearance provides more accurate predictions of human PK compared to alternative scaling approaches (16, 23, 24). As shown in Fig. 4, the simulation results suggest that intensive treatment with Nb_457_-Nb_HSA_-Nb_457_ may lead to slight drug accumulation, consistent with findings from clinical trials involving other therapeutics, such as Ibalizumab and Caplacizumab (14, 20). In the control group, the viral load increased sharply, whereas all treatment groups exhibited significantly lower viral loads compared to the control. Notably, increasing dose intensity had a more pronounced effect on HIV-1 inhibition than increasing the total dose amount (Fig. 5). These findings underscore the potential of Nb_457_-Nb_HSA_-Nb_457_ as a promising therapeutic candidate for HIV-1 treatment. Furthermore, the model holds significant value for prevention trials where direct viral challenge studies cannot be conducted, offering a robust tool for optimizing dosing strategies and evaluating therapeutic efficacy.

Despite its strengths, the PK/PD model has several limitations. First, the sample size in the preclinical study was limited to four mice per group. Additionally, the infection model employed a high inoculum and crude sample preparation techniques, which may not accurately reflect clinical transmission scenarios, such as those occurring through blood or sexual contact. Second, Nb_457_-Nb_HSA_-Nb_457_ targets CD4 protein through Nb_457_ while simultaneously harnessing the binding capacity of Nb_HSA_ toward both human and murine serum albumin proteins (7). The interspecies differences in albumin may alter binding kinetics, which was not reflected in the model due to lacking empirical binding parameters. Here, we only scaled CL and V, while all other human CD4-targeted parameters were assumed to be equivalent to those observed in mice. Further investigation and validation in humans is warranted. Furthermore, due to data sparsity, several assumptions were required to construct the PK-PD model. For instance, HIV-1 infection has been reported to downregulate CD4 expression, likely through the HIV-1 virulence factor nef (25, 26). In patients with chronic HIV-1 infection or late-stage AIDS, reduced CD4 availability may lead to decreased target-mediated clearance of Nb_457_-Nb_HSA_-Nb_457_. Consequently, these patients may require lower doses or less frequent dosing than the projected human regimen. Finally, the extrapolation of preclinical findings to humans remains preliminary due to the lack of human data for validation. For instance, the limited availability of PD viral dynamic data has impeded the validation of PD extrapolation. However, once more clinical *in vivo* data become available, the mechanistic TMDD PK-PD model can be further refined and updated. This will enable its prospective application to simulate unstudied scenarios, aligning with model-informed drug development strategy and providing a robust foundation to support Nb_457_-Nb_HSA_-Nb_457_ in future clinical studies. Moreover, future studies should focus on evaluating the drug’s effects on viral growth dynamics and replication sustainability to refine model parameters and maximize the utility of the PK/PD model for clinical applications.

In conclusion, we developed a mechanism-based TMDD PK-PD model for the anti-CD4 nanobody Nb_457_-Nb_HSA_-Nb_457_, which successfully characterized its PK and HIV-1 viral dynamics, as demonstrated by its ability to reproduce observed PK-PD data in mice. Additionally, the model was scaled from mice to humans to predict potential dosing regimens. This framework provides a valuable tool to support the clinical development of Nb_457_-Nb_HSA_-Nb_457_ by enabling simulations of various dosing strategies to evaluate its efficacy and safety.

## AFTER EPUB

*[This article was published on 19 September 2025 with an error in Acknowledgments. The acknowledgments were corrected in the current version, posted on 4 November 2025.]*

## SUPPLEMENTAL MATERIAL

Supplemental material spectrum.00805-25-s0001.docx Tables S1 and S2; Fig. S1 to S8.

ASM does not own the copyrights to Supplemental Material that may be linked to, or accessed through, an article. The authors have granted ASM a non-exclusive, world-wide license to publish the Supplemental Material files. Please contact the corresponding author directly for reuse.
