# Supplementary material for: Pharmacokinetic-pharmacodynamic modeling of a highly potent and broadly neutralizing anti-CD4 trimeric nanobody to inhibit HIV-1 infection
Source: Microbiol Spectr. 2025 Sep 19;13(11):e00805-25. doi: 10.1128/spectrum.00805-25 (PMC12584636; doi:10.1128/spectrum.00805-25)
Supplement: Supplemental material — Tables S1 and S2; Fig. S1 to S8. [file spectrum.00805-25-s0001.docx]

**Table S1** Summary of the data used to develop the TMDD PK-PD model. The data was derived from our previous study[7].

| **Drug** | **Species** | **Dosing regimen** | **Measurements** |
| --- | --- | --- | --- |
| Nb_457_-Nb_HSA_-Nb_457_ | Mouse | Day 0: HIV-1_CH058_ challenge  Day 1: 400 μg IP  Day 3: 400 μg SC  Day 5: 400 μg SC  Day 7: 400 μg SC | PK: 0, 1, 4, 8, 12, 24, 72, and 120 hours after the dose.  PD: weeks 1, 2, 3, and 4 post-infection. |
| Ibalizumab | Mouse | Day 0: HIV-1_CH058_ challenge  Day 1: 400 μg IP  Day 3: 400 μg SC  Day 5: 400 μg SC  Day 7: 400 μg SC | PK: 0, 1, 4, 8, 12, 24, 72, and 120 hours after the dose.  PD: weeks 1, 2, 3, and 4 post-infection. |

Notes: IP = intraperitoneal; SC = subcutaneous.

**Table S2** Summary of the data used to validate the extrapolated TMDD PK model of Ibalizumab. The data was derived from previous study[14].

| **Drug** | **Species** | **Dosing regimen** | **Measurements** |
| --- | --- | --- | --- |
| Ibalizumab | Human | 10 mg/kg: weekly 10 mg/kg IV infusions for the first 9 doses, followed by 10 mg/kg infusions q2wk;  15 mg/kg: alternating weekly 15 mg/kg IV infusions and placebo for the first 9 doses (up to week 8), followed by 15 mg/kg infusions q2wk;  Placebo: weekly placebo IV infusions for the first 9 doses, followed by placebo infusions q2wk. | PK: day 1 (before the start of therapy), weekly through until week 4, and then q2wk through week 48. Trough PK samples were collected at all visits starting at day 1. |

Notes: IV = intravenous.

**
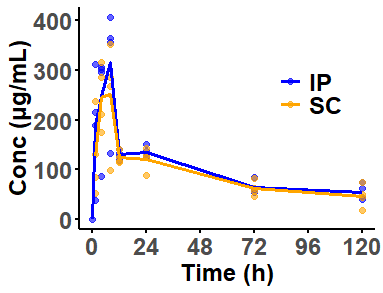
Figure S1**. Time course profiles of Ibalizumab serum concentrations after IP or SC injections of 400 μg.


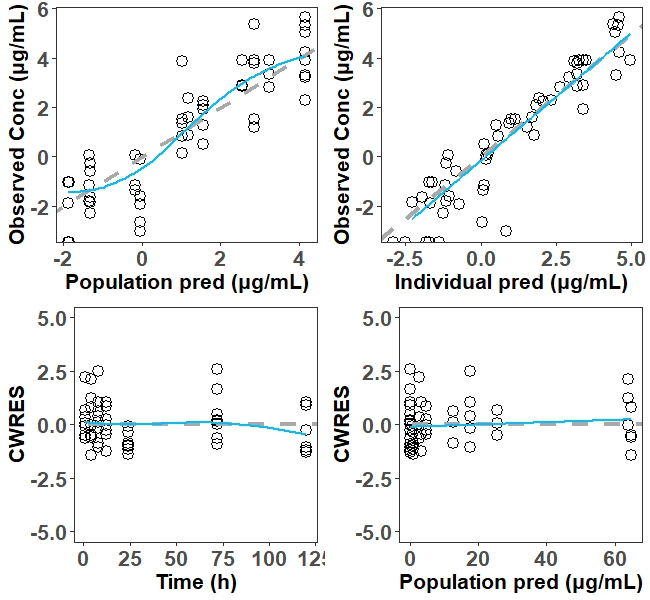


**Figure S2**. Goodness-of-fit (GOF) diagnostic plots of Nb_457_-Nb_HSA_-Nb_457_ final PK model. The top panels present the log-transformed observed data vs. log-transformed population predictions (left), and the log-transformed observed data vs. log-transformed individual predictions (right). The bottom panels present the conditional weighted residual (CWRES) vs. time (left) and log-transformed population predictions (right), respectively. The blue lines are the loess smooth lines. The gray diagonal (top panels) and horizontal (bottom panels) lines are the identity and zero lines, respectively.


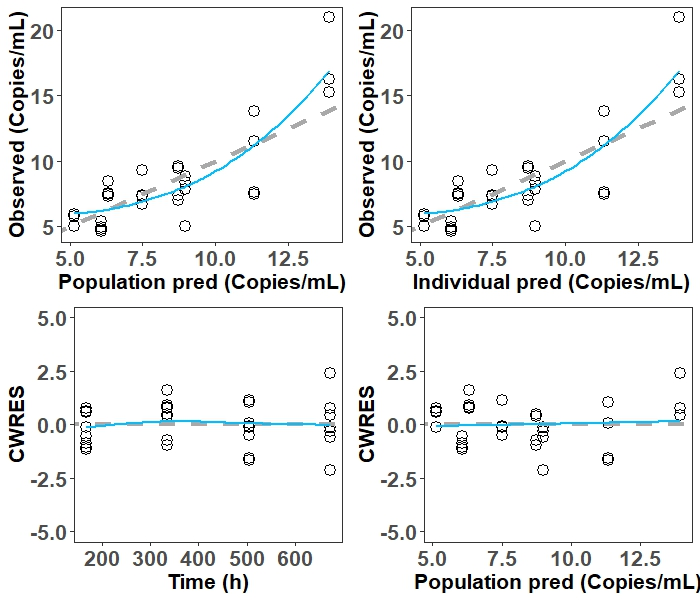

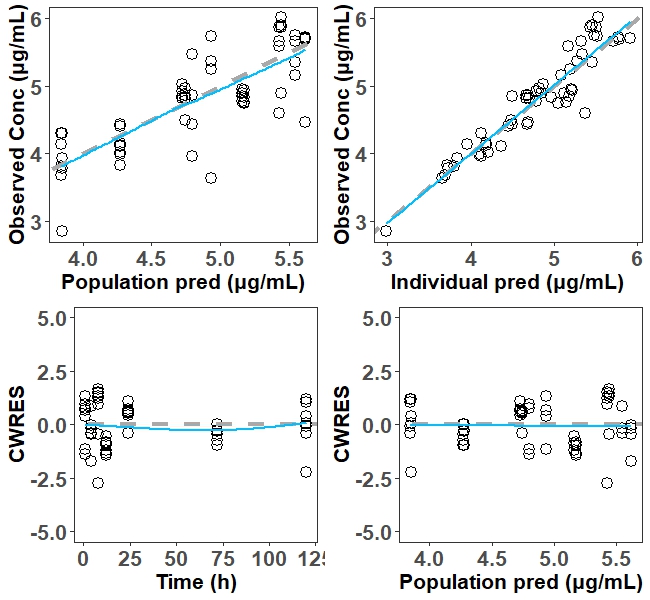
**Figure S3**. Goodness-of-fit (GOF) diagnostic plots of Ibalizumab final PK model. The top panels present the log-transformed observed data vs. log-transformed population predictions (left), and the log-transformed observed data vs. log-transformed individual predictions (right). The bottom panels present the conditional weighted residual (CWRES) vs. time (left) and log-transformed population predictions (right), respectively. The blue lines are the loess smooth lines. The gray diagonal (top panels) and horizontal (bottom panels) lines are the identity and zero lines, respectively.

**Figure S4**. Goodness-of-fit (GOF) diagnostic plots of Nb_457_-Nb_HSA_-Nb_457_ final PD model. The top panels present the log-transformed observed data vs. log-transformed population predictions (left), and the log-transformed observed data vs. log-transformed individual predictions (right). The bottom panels present the conditional weighted residual (CWRES) vs. time (left) and log-transformed population predictions (right), respectively. The blue lines are the loess smooth lines. The gray diagonal (top panels) and horizontal (bottom panels) lines are the identity and zero lines, respectively.


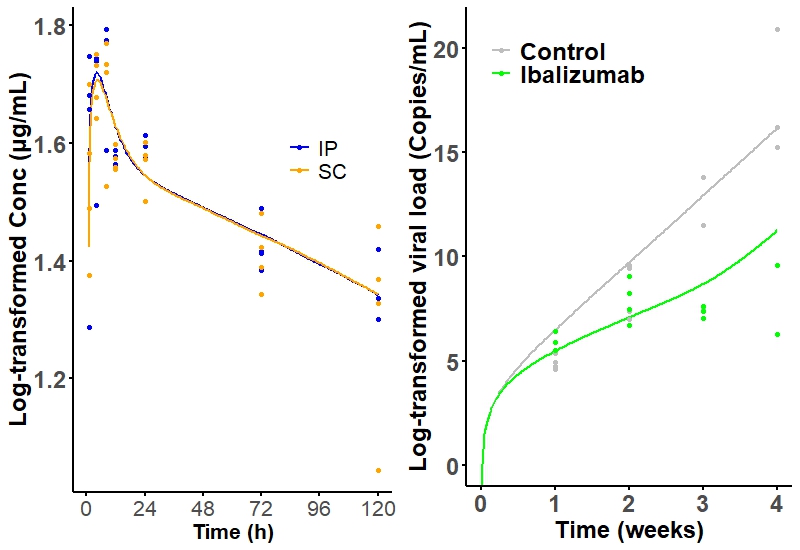

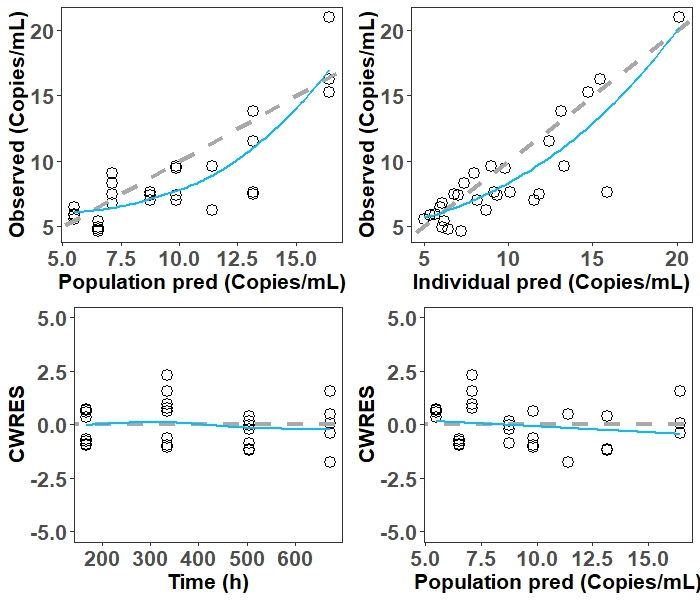
**Figure S5**. Goodness-of-fit (GOF) diagnostic plots of Ibalizumab final PD model. The top panels present the log-transformed observed data vs. log-transformed population predictions (left), and the log-transformed observed data vs. log-transformed individual predictions (right). The bottom panels present the conditional weighted residual (CWRES) vs. time (left) and log-transformed population predictions (right), respectively. The blue lines are the loess smooth lines. The gray diagonal (top panels) and horizontal (bottom panels) lines are the identity and zero lines, respectively.

**Figure S6**. Visual predictive checks (VPC) for the Ibalizumab PK (left) and PD model (right). The solid lines represent the median of the model predictions, and the dots represent the observed data.


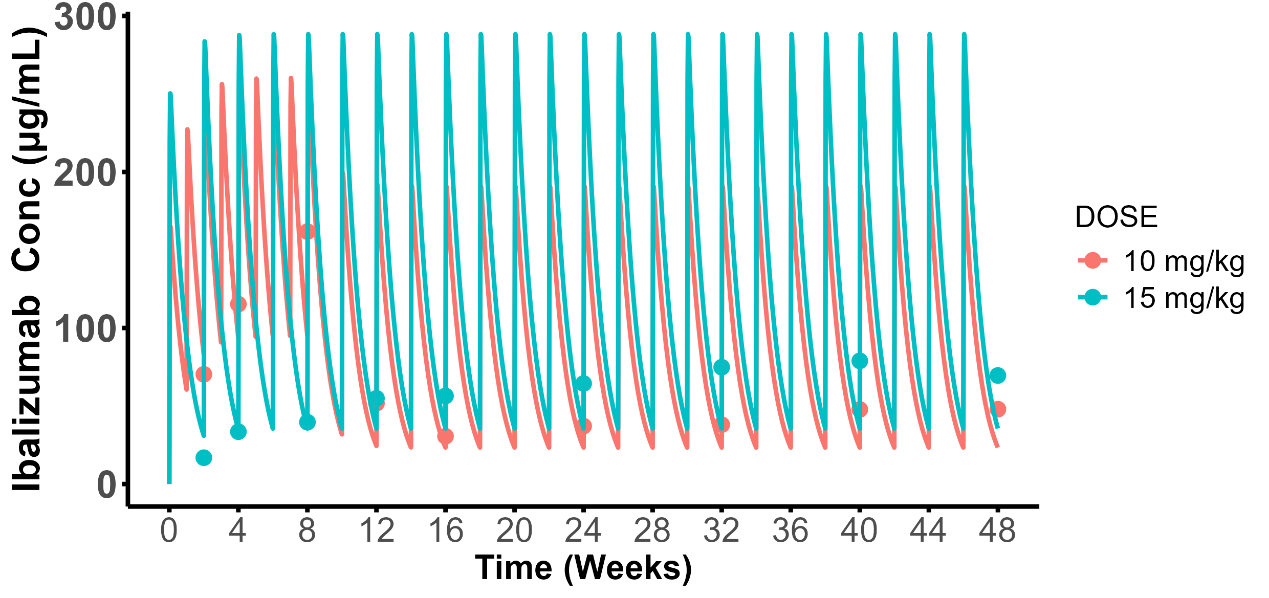
**Figure S7**. Observed and predicted PK profiles for Ibalizumab in human. Solid lines represent model predicted results, and symbols indicate observed data points. Different colors represent different dosing regimens.


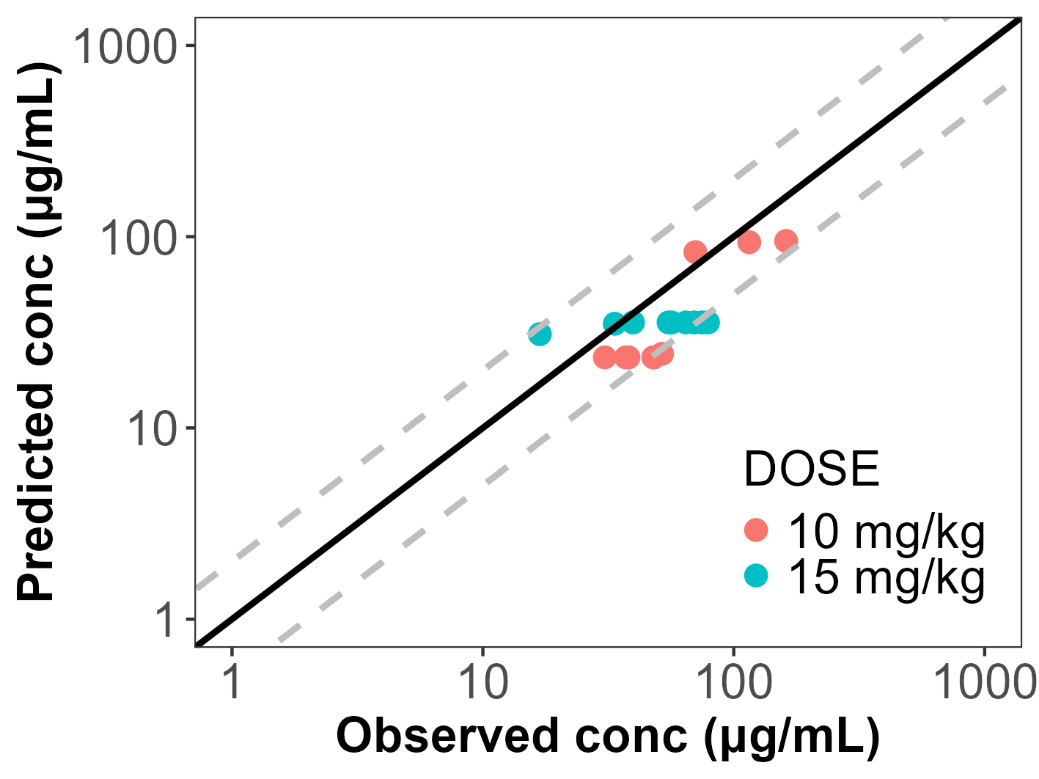


**Figure S8**. The correlation between observed and predicted plots demonstrates the predictive performances of PK of Ibalizumab in human. The solid thin line indicates unity, while dashed lines on either side of the unity line represent two-fold deviations. Different colors denote different dosing regimens.
